# Trauma deaths of hospitalized patients in Abu Dhabi Emirate: a retrospective descriptive study

**DOI:** 10.1186/s13017-023-00501-y

**Published:** 2023-04-28

**Authors:** David O. Alao, Arif Alper Cevik, Fikri M. Abu-Zidan

**Affiliations:** 1 Department of Internal Medicine, Section of Emergency Medicine, College of Medicine and Health Sciences, United Arab Emirates University, Al-Ain, United Arab Emirates; 2 The Department of Emergency Medicine, Tawam Hospital, Al-Ain, United Arab Emirates; 3 The Research Office, College of Medicine and Health Sciences, United Arab Emirates University, Al-Ain, United Arab Emirates

**Keywords:** Major trauma, In-hospital deaths, Location of death, Mode of death

## Abstract

**Aim:**

To study the epidemiology and pattern of trauma-related deaths of hospitalized patients in Abu Dhabi Emirate, United Arab Emirates, in order to improve trauma management and injury prevention.

**Methods:**

The Abu Dhabi Trauma Registry prospectively collects data of all hospitalized trauma patients from seven major trauma centres in Abu Dhabi Emirate. We studied all patients who died on arrival or after admission to these hospitals from January 2014 to December 2019.

**Results:**

There were 453 deaths constituting 13.5% of all trauma deaths in the Abu Dhabi Emirate. The median (IQR) age of the patients was 33 (25–45) years, and 82% were males. 85% of the deaths occurred in the emergency department (ED) and the intensive care unit (ICU). Motor vehicle collision (63.8%) was the leading cause of death. 45.5% of the patients had head injury. Two of the seven hospitals admitted around 50% of all patients but accounted for only 25.8% of the total deaths (*p* < 0.001). Those who died in the ward (7%) were significantly older, median (IQR) age: of 65.5 (31.75–82.25) years, (*p* < 0.001), 34.4% of them were females (*p* = 0.09). The median (IQR) GCS of those who died in the ward was 15 (5.75–15) compared with 3 (3–3) for those who died in ED and ICU (*P* < 0.001).

**Conclusions:**

Death from trauma predominantly affects young males with motor traffic collision as the leading cause. Over 85% of in-hospital deaths occur in the ICU and ED, mainly from head injuries. Injury prevention of traffic collisions through enforcement of law and improved hospital care in the ED and ICU will reduce trauma death.

**Supplementary Information:**

The online version contains supplementary material available at 10.1186/s13017-023-00501-y.

## Introduction

Major trauma is the leading cause of death for ages below 44 years [1]. Annually, 4.4 million people die, and tens of millions more have trauma-related disabilities globally [2]. In 2017, transport injuries, self-harm injuries, and interpersonal injuries accounted for more than 1.3 million deaths each, while falls was responsible for over 695,000 deaths [3]. Between 2007 and 2017, deaths from injuries increased by 2.3%, while death rates were reduced by 13.7% [3]. This improved trend varied globally. Death rates of traffic injuries remained unchanged in South Asia and Southern Latin America over the same time, while other regions experienced a reduction [4]. The health burden of injury is far higher in low and middle-income countries [2].

The United Arab Emirates (UAE), a developing country, has one of the highest GDPs per capita in the world [5]. It has experienced rapid infrastructural development in the last three decades, with a significant increase in civil construction work and transportation [6]. These developments were associated with an increased incidence of injuries from road traffic collisions and falls. Injury is now the second leading cause of death in the UAE [7]. There is a paucity of studies on trauma-related deaths in the UAE. Previous studies have focused mainly on specific patient groups [8–10].

In 2014, a trauma registry was established by the Department of Health (DOH) of Abu Dhabi to assess the magnitude and trends of major trauma in the Emirates. Studying the epidemiology and pattern of trauma deaths will provide information that can be used for health policy planning and injury prevention interventions, which may further reduce trauma deaths in our setting. We aimed to study the epidemiology and pattern of trauma-related deaths of hospitalized patients in Abu Dhabi Emirate, United Arab Emirates, in order to improve trauma management and injury prevention.

## Patients and methods

### Ethical considerations

This study was approved by the Department of Health (DOH) of the Abu Dhabi Emirate. (Ref: DOH/CVDC/2021/932). The patients or their caregivers gave their written informed consent to use their anonymous data for research.

### Study design

This is a retrospective descriptive study of all patients who died in the major trauma centres of Abu Dhabi Emirate, United Arab Emirates, over 6 years. Although the analysis of the data was retrospective, the data were prospectively collected as part of the Abu Dhabi Trauma Registry.

### Setting

Abu Dhabi Emirate, which has three regions, had a population of 2.91 million in 2016. Out of which, 1,807,000 were in the Abu Dhabi region, 766,900 were in the Al-Ain region, and 334,000 were in the western region of Abu Dhabi [11].

### The Abu Dhabi Trauma Registry

**Fig. 1:**
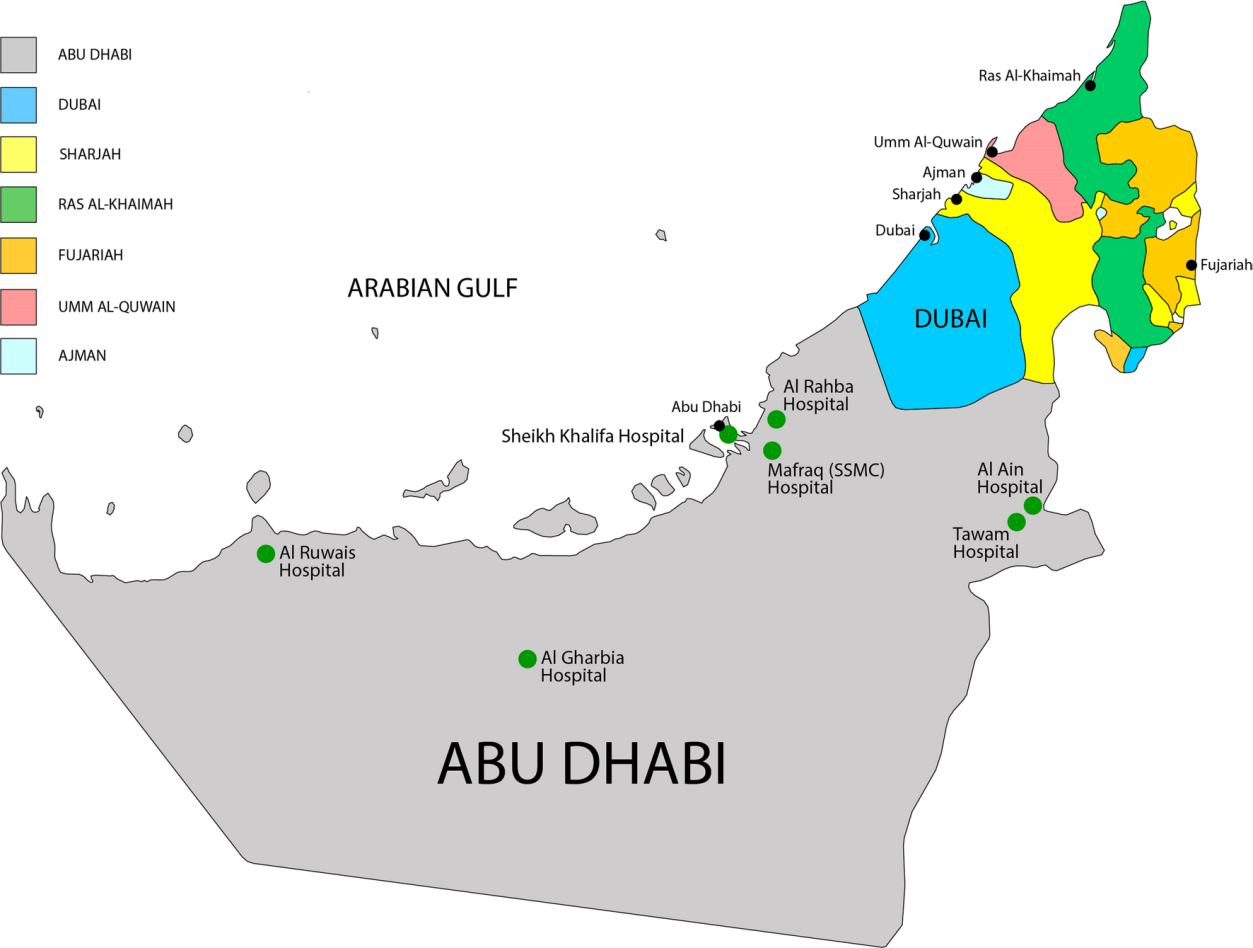
A map showing the locations of the seven contributing hospitals to the Abu Dhabi Trauma Registry in Abu Dhabi Emirate, United Arab Emirates during the period of 2014–2019

This registry was started in 2014 by a legislative order of the Department of Health. The registry was based on the American College of Surgeons' National Trauma Data Bank dataset. It prospectively collects data on all trauma patients who die in the hospital or are admitted to the hospital. There are seven contributing hospitals—three from the Abu Dhabi City region (Mafraq, Khalifa Medical Centre and Al Rahba Hospitals), two hospitals from the Al Ain City region (Tawam and Al Ain Hospitals) and two hospitals from the Al Dhafra Region (Al Gharbia and Al Ruwais) (Fig. 1). Trained registry nurses enter the data. The two community-based teaching hospitals in Al Ain City serve a population of 760,000 inhabitants in the Al Ain region of Abu Dhabi Emirates, the United Arab Emirates [12]. Each hospital has 450 in-patient beds with a full tertiary service of neurosurgery, orthopaedics, general surgery and critical care. In the Abu Dhabi region, the two hospitals, Sheikh Khalifa Medical City (SKMC) hospital and Mafraq Hospital, have a bed base of 450 each and complete tertiary services, including critical care, neurosurgery, orthopaedic and general surgery. The latter hospital was closed in January 2020 and was replaced by an ultra-modern 741-bed hospital—the Sheikh Shakhbout Medical City (SSMC) Abu Dhabi—in the same location. The third hospital in Abu Dhabi region, Al Rahba, has 166 beds and provides secondary-level care. The two hospitals in the Al Dhafra region, Al Gharbia (150 beds) and Al Ruwais (132 beds), also provide secondary-level care. All the hospitals are managed by the Department of Health of Abu Dhabi (DOH) with similar health policies like the need to establish a hospital based-trauma registry and appoint a trauma nurse to enter the data of the injured patients. All medical staff who are involved in trauma management as part of trauma teams are required to be ATLS certified. The certificate should be valid and regularly renewed as needed. The patients would be screened by Focussed Assessment Sonography for trauma as a routine, then transferred to have a whole body 64-slice CT scan using a trauma protocol if needed. In principle, the patients were managed according to the standard ATLS guidelines in the Emergency Department. Management of the admitted patients would then vary depending on the available facilities, surgical sub-specialists, and the local policies of each hospital.

### Study population

We included in the study all trauma patients who died on arrival or after admission to the hospital during the period of January 2014 to December 2019.

### Studied variables

These included patients' demographics, initial Emergency Department (ED) physiological parameters, Glasgow Coma Scale (GCS), Injury Severity Score (ISS), location of death, time of death, mechanism of injury, location of injury, ICU and hospital length of stay, ED discharge destination, and total length of stay in ED.

### Statistical analysis

Data were presented as median (IQR) or number (%) as appropriate. Patients were divided into four groups depending on the hospital location where they died: The ED, the operating theatre, the ICU, and the ward. The groups were compared using the Kruskal–Wallis's test for continuous or ordinal data for more than two groups and Fisher's Exact test or Pearson's Chi-Square for categorical data as appropriate. The IBM SPSS Statistics Program version 28 (SPSS Inc, Chicago, IL, USA) was used to analyze the data with a *p*-value of less than 0.05 to be accepted as statistically significant.

## Results

**Fig. 2:**
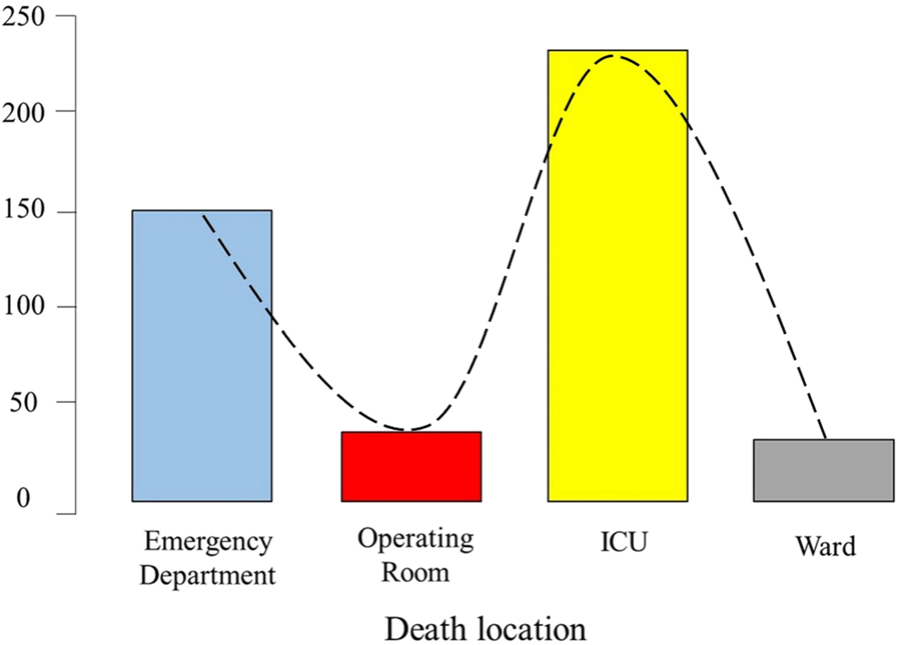
Bar chart of the location of death of hospitalized trauma patients in the Emirate of Abu Dhabi, United Arab Emirates during the period of January 2014 to December 2019, *n* = 453 according to the Abu Dhabi Emirate Trauma Registry

Over the study period, data from 30,239 hospitalized trauma patients were entered into the registry. Twenty-nine thousand seven hundred nine patients (98.2%) survived; 453 died (1,5%), while the outcome was unknown in 77 (0.25%) patients. Over the six-year study period, an average of 76 patients died per year, with an incidence of 2.6 per 100 000 population [10]. There were 562 injury deaths in the Abu Dhabi Emirate in 2017 [11], indicating that deaths of hospitalized trauma patients constituted only 13.5% of all deaths, with most deaths occurring in the prehospital setting. Figure 2 shows the location where the death occurred. The majority (85%) occurred in the ICU (51.7%) and the ED (33.3%). Only 15% occurred in the operating theatre and wards, with almost an equal distribution between them.

Table 1 demonstrates the demography of the trauma deaths. A majority (82%) were males. RTC was the leading cause of death (63.8%), followed by fall from height (11.3%), fall on the same level (7.7%) and assault (7.1%). Those who died in the wards were significantly older (*p* < 0.001), double the age compared with the other locations, tended to be a female (*p* = 0.09), a percentage of more than double those in other locations, with a large proportion of UAE nationals (43.8%, *p* = 0.046), and a significantly higher number of fall from the same level (50%, *p* < 0.001). A significant number of those who died in the ward were transported to the hospital using private cars (*p* < 0.001). There was no significant difference in the injury place between the four groups of dead patients. There were significant differences in the location of death between the hospitals where the death occurred (*p* < 0.001). The majority of deaths in the ED occurred in Hospital B (31.8%); the majority of deaths in the operating room occurred in Hospital A (38.9%); the majority of deaths in the ICU (43.2%), and in the wards (43.8%) occurred in Hospital C.

**Table 1:** Demography by death place of hospitalized trauma patients in Abu Dhabi Emirate, United Arab Emirates, during the period of January 2014 to December 2019 (*n* = 453)

Variable	Overall *n* = 453	Emergency room *n* = 151	Operating room *n* = 36	ICU *n* = 234	Ward *n* = 32	*P* value
Age	33 (25–45)	32 (23–42)	31 (23–45)	32 (25–43)	65.50 (31.75–82.25)	< 0.001
Gender						0.09
Female	82 (18.1)	22 (14.6)	6 (16.7)	43 (18.4)	11 (34.4)	
Male	371 (81.9)	129 (85.4)	30 (83.3)	191 (81.6)	21 (65.6)	
Nationality						0.046
UAE	123 (27.2)	46 (30.5)	6 (16.7)	57 (24.4)	14 (43.8)	
Non-UAE	330 (72.8)	105 (69.5)	30 (83.3)	177 (75.6)	18 (56.3)	
Hospital						< 0.001
A (*n* = 7210)	57 (12.6)	16 (10.6)	14 (38.9)	21 (9.0)	6 (18.8)	
B (*n* = 4136)	98 (21.6)	48 (31.8)	3 (8.3)	39 (16.7)	8 (25.0)	
C (*n* = 5050)	135 (29.8)	19 (12.6)	1 (2.8)	101 (43.2)	14 (43.8)	
D (*n* = 3630)	74 (16.3)	23 (15.2)	11 (30.6)	37 (15.8)	3 (9.4)	
E (*n* = 7015)	60 (13.2)	34 (22.5)	6 (16.7)	20 (8.5)	0 (0.0)	
F (*n* = 1659)	20 (4.4)	4 (2.6)	1 (2.8)	14 (6.0)	1 (3.1)	
G (*n* = 319)	9 (2.0)	7 (4.6)	0 (0.0)	2 (0.9)	0 (0.0)	
Transportation						< 0.001
Ambulance	408 (90.1%)	131 (86.8)	33 (91.7)	222 (94.9)	22 (68.8)	
Private car	45 (9.9)	20 (13.2)	3 (8.3)	12 (5.1)	10 (31.3)	
Injury place						0.67
Public roads	131 (39.2)	32 (31.4)	14 (43.8)	78 (44.1)	7 (30.4)	
Home	113 (33.8)	41 (40.2)	10 (31.3)	55 (31.1)	7 (30.4)	
Work	48 (14.4)	12 (11.8)	6 (18.8)	24 (13.6)	6 (26.1)	
Public areas	30 (9.0)	11 (10.8)	2 (6.3)	15 (8.5)	2 (8.7)	
Desert/sea/water	10 (3.0)	5 (4.9)	0 (0.0)	4 (2.3)	1 (4.3)	
Farm	2 (0.6)	1 (1)	0 (0.0)	1 (0.6)	0 (0.0)	
Mechanism of injury						< 0.001
MVC	289 (63.8%)	114 (75.5)	21 (58.3)	150 (64.1)	4 (12.5)	
Fall from height	51 (11.3)	16 (10.6)	9 (25.0)	24 (10.3)	2 (6.3)	
Fall same level	35 (7.7)	3 (2.0)	1 (2.8)	15 (6.4)	16 (50.0)	
Falling object	18 (4.0)	4 (2.6)	1 (2.8)	12 (5.1)	1 (3.1)	
Machinery	3 (0.7)	0 (0)	1 (2.8)	2 (0.9)	0 (0.0)	
Assault	32 (7.1)	9 (6.0)	3 (8.3)	19 (8.1)	1 (3.1)	
Burn	25 (5.5)	5 (3.3)	0 (0.0)	12 (5.1)	8 (25)	

Continuous and ordinal data are presented as median (25–75 IQR) while categorical data are presented as number (%)

*P* = Kruskal–Wallis test, Fisher’s Exact test or Pearson Chi Square as appropriate

Percentages are calculated from valid numbers excluding missing data. Number may not add to 453 because of missing data

Table 2 shows that the maximum injured regions were significantly different between the patients depending on their death place. The head/brain occurred in 45.5% of the patients and was higher in those who died in the ED (50.3%) and the ICU (49.6%). Chest injuries were higher in those who died in the ED (17.2%). Those who had two body regions injured were higher in those who died in the operating room (30.6%) and the ICU (30.8%).

**Table 2:** Maximum injured region by death place of hospitalized trauma patients in Abu Dhabi Emirate, United Arab Emirates, during the period of January 2014 to December 2019 (*n* = 453)

Region	Overall *n* = 453	Emergency room *n* = 151	Operating room *n* = 36	ICU *n* = 234	Ward *n* = 32	*P* value
						< 0.001
Head/brain	206 (45.5)	76 (50.3)	11 (30.6)	116 (49.6)	3 (9.4)	
Chest/lungs	35 (7.7)	26 (17.2)	4 (11.1)	5 (2.1)	0 (0.00)	
Abdomen	8 (1.8)	4 (2.6)	1 (2.8)	3 (1.3)	0 (0.00)	
Pelvis	10 (2.2)	4 (2.6)	1 (2.8)	4 (1.7)	1 (3.1)	
Spine	8 (1.8)	3 (2.0)	1 (2.8)	1 (0.4)	3 (9.4)	
Extremities	21 (4.6)	5 (3.3)	2 (5.6)	3 (1.3)	11 (34.4)	
2 body regions	112 (24.7)	23 (15.2)	11 (30.6)	72 (30.8)	6 (18.8)	
3 or more body regions	27 (6.0)	3 (2.0)	5 (13.9)	19 (8.1)	0 (0.00)	
Not specified	26 (5.7)	7 (4.6)	0 (0.00)	11 (4.7)	8 (25.00)	
Total	453 (100)	151 (100)	36 (100)	234 (100)	32 (100)	

Data are presented as number (%)

*P* = Fisher’s Exact test or Pearson Chi Square as appropriate

Percentages are calculated from valid numbers excluding missing data. Number may not add to 453 because of missing data

**Fig. 3:**
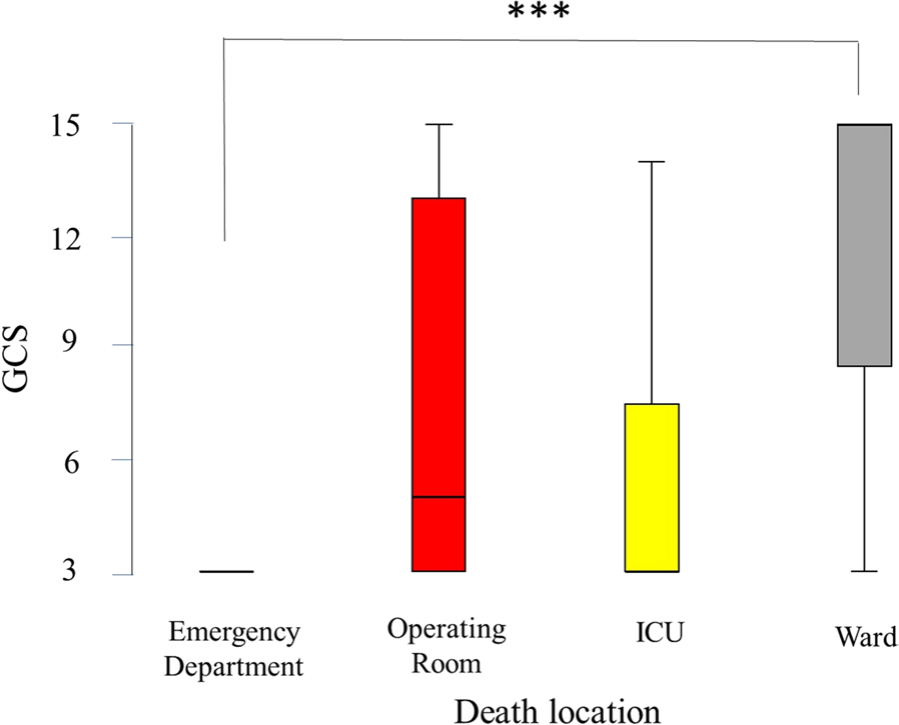
Box-and-whisker plot of Glasgow Coma Scale (GCS) of the hospitalized trauma patients in the Emirate of Abu Dhabi, United Arab Emirates by location of death, who died during the period of January 2014 to December 2019, *n* = 453, according to the Abu Dhabi Emirate Trauma Registry. The box represents the 25th to the 75th percentile IQR. The horizontal line within each box represents the median. ***p < 0.001, Kruskal–Wallis test

**Fig. 4:**
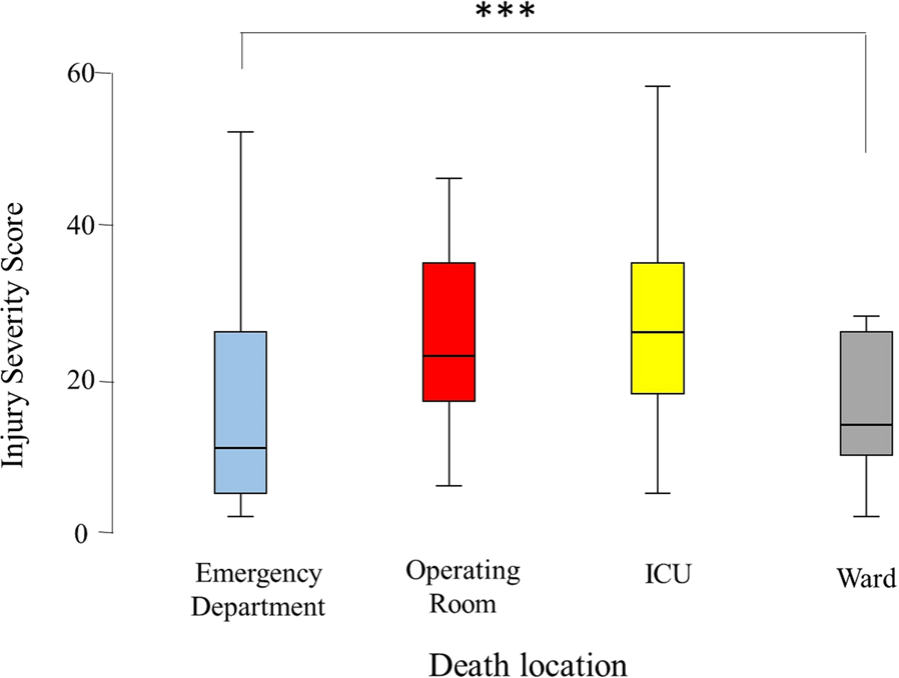
Box-and-whisker plot of Injury Severity Score of the hospitalized trauma patients in the Emirate of Abu Dhabi, United Arab Emirates by location of death, who died during the period of January 2014 to December 2019, *n* = 453, according to the Abu Dhabi Emirate Trauma Registry. The box represents the 25th to the 75th percentile IQR. The horizontal line within each box represents the median. ***p < 0.001, Kruskal–Wallis test

Table 3 shows the severity markers by death place, including systolic blood pressure, pulse per minute, respiratory rate, GCS, trauma team response, time in the ED, ventilation time, ISS, ICU stay, and hospital stay. All these markers were significantly different between the four groups of patients (*P* < 0.001 in each). Those who were declared dead in the ED were mainly without systolic blood pressure and pulse. Those who died in the ward had a median SBP of 130 mmHg. Those who died in the ED and ICU had a median GCS of 3, while those who died in the ward had a median GCS of 15 (Fig. 3). Those who died in the operating room and ICU had significantly higher ISS (almost double) compared with those who died in the ED and ward (Table 3; Fig. 4).

**Table 3:** Severity markers by death place of hospitalized trauma patients in Abu Dhabi Emirate, United Arab Emirates, during the period of January 2014 to December 2019 (*n* = 453)

Variable	Overall *n* = 453	Emergency room *n* = 151	Operating room *n* = 36	ICU *n* = 234	Ward *n* = 32	*P* value
SBP	108 (0–140)	00 (00–00)	104.50 (78.25–135.00)	121.00 (98–148)	130 (102.25–146)	< 0.001
Pulse	88 (35–118)	00 (00–15)	104.50 (77.25–123.75)	102 (78–124.50)	91.50 (80.50–116.75)	< 0.001
RR	18 (00–22.25)	00 (00–00)	20 (15.50–25.50)	20 (16–24)	18 (18–22)	< 0.001
GCS	3 (3–7)	3 (3–3)	5 (3–13)	3 (3–8)	15 (5.75–15)	< 0.001
Response level						< 0.001
No activation	16 (3.5)	7 (4.6)	0 (0.00)	8 (3.4)	1 (3.1)	
Only consultation	119 (26.3)	40 (26.5)	4 (11.1)	53 (22.6)	22 (68.8)	
Full code	312 (68.9)	102 (67.5)	32 (88.9)	170 (72.6)	8 (25.0)	
Time in ER	138 (70–222)	97 (21–195.5)	118.50 (69.5–171.50)	156 (100–231)	223.50 (00–344.25)	< 0.001
Ventilation time	2 (0–11)	0 (0–1)	2.5 (1.00–8.75)	9 (3–17)	2.50 (00–7.00)	< 0.001
ISS	22 (10–33)	10 (4–25)	22 (16–34)	25 (17–34)	13.00 (9.00–25.00	< 0.001
ICU stay	3 (0–11)	0 (00–00)	3 (00–10.50)	9.00 (3.75–17.00)	6.00 (1.25–18.50)	< 0.001
Hospital stay	2 (1–11)	1.00 (1.00–1.00)	2 (1.00–9.50)	8.50 (3.00–16.00)	6.00 (3.25–23.25)	< 0.001

Continuous and ordinal data are presented as median (25–75 IQR) while categorical data are presented as number (%)

*P* = Kruskal–Wallis test, Fisher’s Exact test or Pearson Chi Square as appropriate

Percentages are calculated from valid numbers excluding missing data. Number may not add to 453 because of missing data

**Fig. 5:**
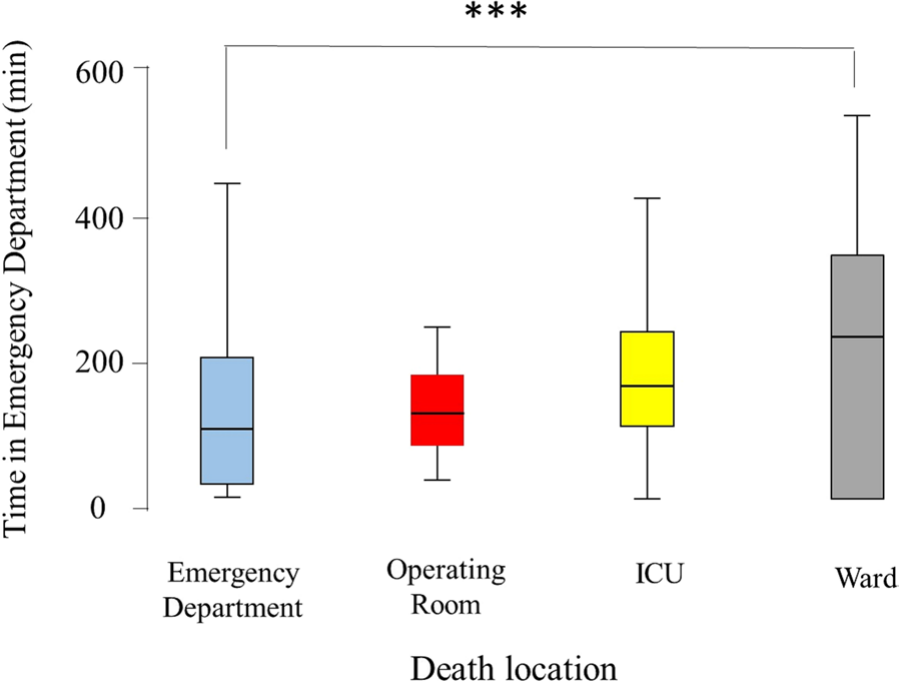
Box-and-whisker plot of time in the Emergency Department in minutes of the hospitalized trauma patients in the Emirate of Abu Dhabi, United Arab Emirates by location of death, who died during the period of January 2014 to December 2019, *n* = 453, according to the Abu Dhabi Emirate Trauma Registry. The box represents the 25th to the 75th percentile IQR. The horizontal line within each box represents the median. ***p < 0.001, Kruskal–Wallis test

**Fig. 6:**
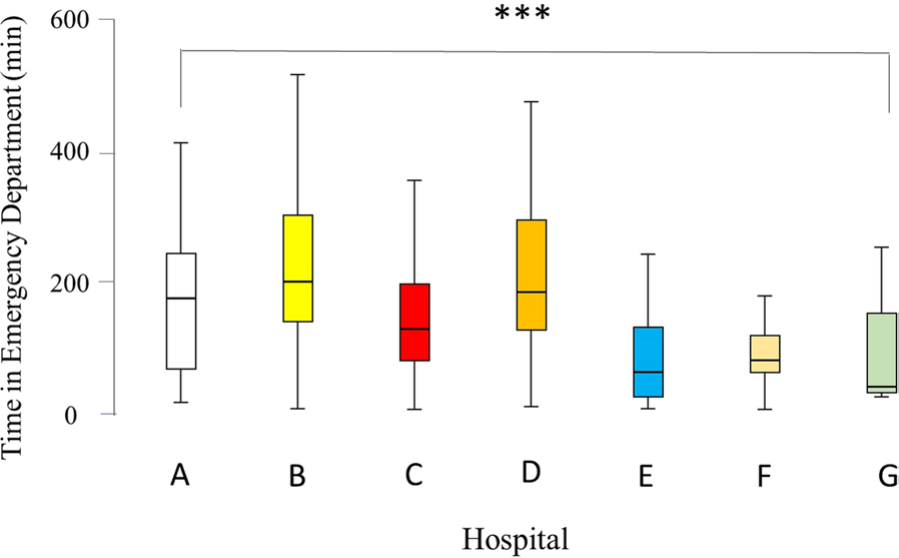
Box-and-whisker plot of time in the Emergency Department in minutes of the hospitalized trauma patients in the Emirate of Abu Dhabi, United Arab Emirates by treating hospital, who died during the period of January 2014 to December 2019, *n* = 453, according to the Abu Dhabi Emirate Trauma Registry. The box represents the 25th to the 75th percentile IQR. The horizontal line within each box represents the median. ***p < 0.001, Kruskal–Wallis test

Time spent in the ED had a median of 138 min. This was significantly different between patients of the four groups (Fig. 5). The time spent in the ED was the longest for those who died in the ward, followed by those who died in the ICU, operating room, and finally the ED. There was a significant difference in the time spent in the ED between the seven hospitals (*p* < 0.001) (Fig. 6).

Table 4 shows the standardized death incidence of hospitalized trauma patients in the three regions of the Abu Dhabi Emirate. The maximum death incidence was in the Al-Ain Region (3.37/100 000 population), followed by the Abu Dhabi City region (2.48/100 000 population) and the Al Dhafra Region (1.45/100 000 population). The median time spent in the ED for those who died in the Al-Ain City Region was 183 min compared with 125 in the Abu Dhabi City Region and 73 in the Western Region. However, ISS in the Al-Ain City Region was significantly less [Median (IQR) 17 (9–28) compared with 25 (13–34) and 25 (17–39)] in the other two regions.

**Table 4:** The standardised death incidence of the hospitalized trauma patients in the three regions of Abu Dhabi Emirate, United Arab Emirates, during the period of January 2014 to December 2019 (*n* = 453)

Variable	Abu Dhabi Region	Al Ain Region	Western Region	*P* value
Trauma deaths over 6 years	269	155	29	
Annual deaths	44.83	25.83	4.83	
Population	1 807 000	766 900	334 000	
Standardised deaths/100 000	2.48	3.37	1.45	
SBP	106 (50–140)	111 (0–138)	90 (0–134)	0.53
GCS	3 (3–7)	3 (3–8)	3 (3–8)	0.98
ISS	25 (13–34)	17 (9–29)	25 (17–39)	0.01
Time in ED (min)	125 (66–202)	183 (104–272)	73 (34–124)	< 0.001

Data were standardised using the population in 2016 (mid period) [Abu Dhabi Health statistics 2017]

## Discussion

This study has shown that hospitalized trauma deaths constitute only 13.5% of all trauma deaths in Abu Dhabi. Eighty-two per cent of the deaths were in young males, and 85% occurred in either the ED or the ICU. Motor vehicle collisions, causing mainly head injuries or injuries to two body regions, and fall from height were the two predominant mechanisms in the patients who died. The in-hospital mortality rate of 1.5% is less than the 4.7% reported in the United States [13] and 8.3% from the UK [14]. The low death rate in this study reflects our registry criteria, which allows for the inclusion of trauma patients with low ISS. However, our median age of 33 years is low compared with the median of 48 years reported from Australia [15] and 64.5 years from UK [14]. This indicates greater years of life lost (YLL) in our setting.

In the suggested trimodal distribution of trauma deaths, over 50% of deaths occur in the first few hours before the patients reach the hospitals [16]. The 86% prehospital death rate in our study is more than the reported range of 30–70% [17]. Furthermore, most of the deaths in the ED were in patients with no vital signs on arrival. These patients could arguably be classified as prehospital deaths, thus further increasing the proportion of those who died in the prehospital setting. The high prehospital death rate may be attributed to the severity of the primary injury or prolonged transfer times. Increased injury severity rate, defined as the ratio of fatalities and injuries per 1000 road traffic accidents was reported in our setting [18]. The current study did not look at the ambulance on-scene and transfer times. However, the geographical distribution of the trauma centers and the land mass of the Abu Dhabi Emirate, which represents 87% of the UAE as shown in Fig. 1, may suggest that delayed transfer time is a contributory factor. This is mitigated by the network of good roads and the interventions provided by our EMS, which are limited to providing airway support and hemorrhage control on the scene. There is a major challenge in preventing the high prehospital deaths in our setting. Pfeifer et al. reported that only 4.9–11.3% of prehospital trauma deaths are preventable. They identified delayed transportation, early treatment, and medical errors as the three main contributors to high prehospital death rates [19]. Helicopters can reduce delays in transfer of trauma patients over long distances, and they are an effective component of a trauma system [20]. Only a small proportion of trauma patients are transferred to the hospital by helicopters in our setting. Intervention measures must focus on these areas to reduce our high prehospital death rates.

Our study has shown two definite in-hospital peaks; one in the ED, which was immediate, and a second in the ICU which occurred over several days. Gunst et al. described a bimodal distribution of deaths, where the first peak occurred in the prehospital phase, followed by a second peak several days later [21]. More recent studies have shown an early peak occurring within hours, followed by a gradual decrease in deaths over time without any peak [22]. Rather than looking at trauma deaths as a function of time [23] because we do not have the prehospital death data, we chose instead to look at the locations in the hospital where the deaths have occurred. This may help to identify where in-hospital intervention measures and resource allocation can be most effective. Our results show the ED (33.3%) and the ICU (51.7%) as the two areas where most of the deaths have occurred and where intervention measures must be directed.

In this study, head injuries accounted for over 45% of the deaths while hemorrhagic deaths (injuries to the abdomen, pelvis and, to some extent, the chest) constituted only 10%. This indicates that head injury remains a major problem in our setting. Earlier deaths from head injuries may have resulted from hypoxia and airway compromise, while the later deaths are most likely caused by severe traumatic brain injuries. Advances in trauma care across the globe have demonstrated a reduction in-hospital trauma deaths due to hemorrhage but little change in death due to traumatic brain injury [24, 25]. Nonetheless, while primary brain injuries can only be mitigated by legislation and primary injury prevention measures, much can be done to prevent secondary brain injuries, such as targeted brain protection from hypoxia, hypercarbia, hypotension, and early neurosurgical intervention. This approach was highlighted in the World Society of Emergency Surgery consensus guidelines on the management of poly-trauma patients with severe head injuries [26]. Adopting these measures, and further development of the existing neuro-intensive care will improve survival in the ICU patients with head injury, which is close to 50% in this study.

About 7% of the reported deaths in this study occurred in the ward. These were mainly elderly patients with limb injuries and normal vital signs on arrival at the hospital. This reflects the population structure of the UAE in which most of the elderly are Emirati while most of the younger population are expatriates who tend to retire to their home countries. The median ED length of stay in these patients was almost double the overall median ED length of stay of two hours. Many studies have reported an increasing trend in trauma in the elderly [27–30]. Recognizing these high-risk patients will help focus on intervention measures such as preventing falls in the elderly and optimizing their care in the ED by reducing the ED length of stay and instituting measures to reduce injury complications.

There were significant differences in mortality rates in our trauma-receiving hospitals. Hospitals A and E each received over seven thousand trauma cases during the study period. This equates to more than 1000 trauma admissions per year. The two hospitals admitted close to 50% of all trauma cases but accounted for only 25.8% of the total deaths. In contrast, three other hospitals received between three to five thousand cases representing 40% of the total trauma admission but accounted for almost 70% of the total deaths. There are different factors that may explain this finding, including the annual number of treated trauma patients and the proportion of those with ISS > 15 (Additional file 1: Table S1). Rural hospitals lack angioembolization facilities, some lack neurosurgical care, and they vary in developing their own trauma team structure which depends on the local policies of each hospital. This was reflected in the significant variation of the time spent in the ED between the hospitals. Several studies have shown that the regionalization of trauma services leads to better patient outcomes by concentrating trauma resources and expertise in a few centers [31–33]. The optimal annual trauma admissions that are associated with improved survival are estimated to be greater than 240 patients/year for patients with ISS of more than 15. In our setting, only Hospital E is close to this standard with the remaining hospitals receiving less than half the recommended numbers. (Additional file 1: Table S1). High volume leads to better trauma care outcomes [32, 34]. A system of hubs and spokes with agreed procedures with the EMS to bypass smaller hospitals and transfer trauma patients directly to the major trauma centers will ensure adequate patient volume and the maintenance of skills and improved patients' outcome.

About 75% of those who died in the ward received no trauma code or only had a consultation. This suggests a significant level of under-triaging indicating that the patients were not considered sick and were therefore given a low priority. Furthermore, the median (IQR) age for the patients who died on the ward was 65.50. Appropriate triaging of elderly trauma patients is challenging. The American College of Surgeons Committee on Trauma (ACS-CoT) adult triage tool performs poorly in this age group. Many triage tools that modify the physiological thresholds have been developed to improve triage reliability. Still, elderly trauma patients continue to be under-triaged which impacts their clinical outcomes [35–37]. Adopting any of the new elderly trauma triaging tools may improve outcomes in our population.

Deaths from assaults are uncommon in our setting compared with other countries [38]. Only 7.1% of the deaths in this study resulted from assaults. This has implications for the management of patients who arrive at the ED with traumatic cardiac arrest. Majority of these patients are blunt trauma patients who had road traffic collisions or fall from height having a low survival rate [39]. Additional file 2: Table S2 summarizes the proposed recommendations to improve our trauma system.

### Limitations of the study

Our study has several limitations. There was no prehospital data for on-scene and transfer time. In addition, in some cases, the exact cause of death is unknown, as UAE law does not permit routine post-mortem examination. It is also possible that some patients were treated in other small hospitals, but they were not captured in our study because there was no mortality recorded. Finally, we do not have the catchment population for each hospital as they overlap with each other. Accordingly, we combined hospitals in the same regions to calculate the annual incidence of hospitalized death.

## Conclusions

Death from trauma predominantly affects young males with motor traffic collision as the leading cause in our setting. Over 85% of in-hospital deaths occur in the ICU and ED mainly from head injuries. Injury prevention of traffic collisions through enforcement of law and improved hospital care in the ED and ICU will reduce trauma death.

## Supplementary Information

**Additional file 1. Table S1:** Institutions by annual total admissions, ISS>15 and mortality.

**Additional file 2. Table S2:** Problem areas and proposed recommendations.

## Data Availability

There are no additional data available to share with the readers. Data can be shared with the Editor of the Journal if requested.

## Abbreviations

AIS: Abbreviate Injury Scale
DOH: Department of Health
ED: Emergency Department
EMS: Emergency Medical Services
GCS: Glasgow Coma Scale
ICU: Intensive care unit
IQR: Interquartile range
ISS: Injury Severity Score
NISS: New Injury Severity Score
RR: Respiratory rate
RTC: Road traffic collision
SBP: Systolic blood pressure
UAE: United Arab Emirates
WSES: World Society of Emergency Surgery
