# Supplementary material for: Trauma deaths of hospitalized patients in Abu Dhabi Emirate: a retrospective descriptive study
Source: World J Emerg Surg. 2023 Apr 28;18:31. doi: 10.1186/s13017-023-00501-y (PMC10148441; doi:10.1186/s13017-023-00501-y)
Supplement: Supplementary file 1 — Additional file 1. Table S1: Institutions by annual total admissions, ISS>15 and mortality. [file 13017_2023_501_MOESM1_ESM.docx]

**Supplementary Table 1** shows institutions by annual total admissions, ISS>15 and mortality

| Institution | Type | Number/Annum | ISS>15/Annum | Mortality |
| --- | --- | --- | --- | --- |
| A | Tertiary | 1202 | 71 | 0.8% |
| B | Tertiary | 689 | 77 | 2.4% |
| C | Tertiary | 842 | 67 | 1.5% |
| D | Secondary | 605 | 72 | 1.7% |
| E | Tertiary | 1169 | 212 | 1.9% |
| F | Secondary | 276 | 34 | 1.2% |
| G | Secondary | 53 | 6 | 2.8% |
