# Supplementary material for: Trauma deaths of hospitalized patients in Abu Dhabi Emirate: a retrospective descriptive study
Source: World J Emerg Surg. 2023 Apr 28;18:31. doi: 10.1186/s13017-023-00501-y (PMC10148441; doi:10.1186/s13017-023-00501-y)
Supplement: Supplementary file 2 — Additional file 2. Table S2: Problem areas and proposed recommendations. [file 13017_2023_501_MOESM2_ESM.docx]

**Supplementary Table 2** shows problem areas and proposed recommendations

| **Action Area** | **Proposed recommendation** |
| --- | --- |
| Pre-accident | Improved legislation on road safety, including reducing speed limits on trunk roads |
|  | Designation and accreditation of major trauma centres |
|  | Establish a hub and spoke model with agreed bypass and secondary transfer guidelines |
|  | Reducing current major trauma-receiving hospitals to three |
|  | Targeted education to modify risk behaviour in young male road users |
|  | Active prevention of falls in the elderly |
| Pre-hospital |  |
|  | Integrate pre-hospital service into an inclusive trauma network |
|  | Early advanced airway management for head injury patients |
|  | Reduce transfer time by using helicopters |
| ED |  |
|  | Standardize pre-arrival information for trauma patients |
|  | Modify trauma code criteria for elderly patients |
| ICU |  |
|  | Develop neuro-intensive care |
|  | Expand the neurosurgical services |
| Wards |  |
|  | Prevent complications in elderly trauma |
